# A Teleost Fish Model to Understand Hormonal Mechanisms of Non-breeding Territorial Behavior

**DOI:** 10.3389/fendo.2020.00468

**Published:** 2020-07-23

**Authors:** Ana C. Silva, Lucía Zubizarreta, Laura Quintana

**Affiliations:** ^1^Laboratorio de Neurociencias, Facultad de Ciencias, Universidad de la República, Montevideo, Uruguay; ^2^Unidad Bases Neurales de la Conducta, Departamento de Neurofisiología Celular y Molecular, Instituto de Investigaciones Biológicas Clemente Estable, Montevideo, Uruguay; ^3^Laboratorio de Neurofisiología Celular y Sináptica, Departamento de Fisiología, Facultad de Medicina, Universidad de la República, Montevideo, Uruguay

**Keywords:** *Gymnotus omarorum*, non-breeding aggression, fadrozole, natural spacing, estrogen

## Abstract

Aggressive behaviors occurring dissociated from the breeding season encourage the search of non-gonadal underlying regulatory mechanisms. Brain estrogen has been shown to be a key modulator of this behavior in bird and mammal species, and it remains to be understood if this is a common mechanism across vertebrates. This review focuses on the contributions of *Gymnotus omarorum*, the first teleost species in which estrogenic modulation of non-breeding aggression has been demonstrated. *Gymnotus omarorum* displays year-long aggression, which has been well characterized in the non-breeding season. In the natural habitat, territory size is independent of sex and determined by body size. During the breeding season, on the other hand, territory size no longer correlates to body size, but rather to circulating estrogens and gonadosomatic index in females, and 11-ketotestosterone in males. The hormonal mechanisms underlying non-breeding aggression have been explored in dyadic encounters in lab settings. Males and females display robust aggressive contests, whose outcome depends only on body size asymmetry. This agonistic behavior is independent of gonadal hormones and fast acting androgens. Nevertheless, it is dependent on fast acting estrogenic action, as acute aromatase blockers affect aggression engagement, intensity, and outcome. Transcriptomic profiling in the preoptic area region shows non-breeding individuals express aromatase and other steroidogenic enzyme transcripts. This teleost model reveals there is a role of brain estrogen in the control of non-breeding aggression which seems to be common among distant vertebrate species.

## Introduction

The study of territoriality can provide insight into how animals integrate social and environmental cues with their physiological context to produce behavioral responses. Steroid hormones are key in this integration, affecting behavior through the modulation of brain areas belonging to the social behavior network (1–3). Territoriality occurs when animals defend spatially associated resources against competing individuals, and it is frequently mediated by agonistic encounters (4–6). Animals will only defend resources when the benefits exceed the costs of defense, and this is key to understanding how spacing, mating, and social systems have evolved (7). Pioneering studies in the field of behavioral ecology (8) have shown that optimal cost-benefit balance in territorial defense occurs when animals compete for mating opportunities, while the defense of resources unrelated to reproduction is less often observed. Nonetheless, in a few species territorial defense is present year-round (9–11), which may ensure access to foraging areas or protection from predators across seasons (4). Territorial aggression may thus occur in these unconventional cases uncoupled from a breeding physiology and independently from gonadal hormones.

Aggressive behaviors which occur dissociated from the breeding season, encourage the search of non-gonadal underlying regulatory mechanisms (12, 13). Early reports in wild birds established the independence of non-breeding aggression from circulating androgens (14–16). Many studies have shown that aggression may occur when gonads are regressed and even after castration in some species of birds, mammals, reptiles and fish (9, 11, 17–25). In addition, it has also been reported that territorial challenges during the non-breeding season do not affect circulating testosterone (22, 26, 27). Brain estrogens have been shown to have a forefront role in the regulation of non-breeding aggression. This was first postulated by pioneer research showing that estrogens promote aggression in non-breeding song sparrows (18, 28, 29) and in California mice subjected to a short photoperiod (30, 31). Aromatase, which converts androgens into estrogens, is present in brain regions related to aggression, and may display seasonal changes in activity (32, 33). This raises the question: is the role of brain estrogen underlying non-breeding aggression a general strategy across vertebrates?

Fish are an ideal group to approach this question as they are evolutionarily early vertebrates, they display diverse and elaborate social behaviors, brain areas related to social behavior are conserved, and they exhibit extraordinarily high levels of brain aromatase activity (34–38).

This review focuses on the contributions from a teleost fish model on the hormonal modulation of non-breeding territorial behavior, to better understand different mechanisms underlying aggression. South American weakly electric fish of the Order Gymnotiformes, constitute a highly diverse group. They produce electric organ discharges (EOD) that are used for active sensing and communication [reviewed in (39)], and their well-known electrogenic system is composed of discrete nuclei in the brainstem and spinal cord and a peripheral electric organ. This system has been shown to be hormone-sensitive in many of its components frequently producing sexually dimorphic communication signals, making these fish well established models to study steroid action on neural circuits underlying behavior (40–49). *Gymnotus omarorum* occurs naturally at the southern boundary of gymnotiform distribution in South America (Uruguay). It is a seasonal breeder, yet it displays year-round territoriality in both males and females (50). It allows the analysis of territorial aggression in the natural habitat as well as the exploration of its proximate mechanisms in lab settings. The fact that this behavior occurs when gonads are regressed and circulating sex steroids are low, puts the spotlight on brain synthesis of steroid hormones. This is the first teleost model that contributes to revealing common estrogenic roles in the control of non-breeding aggression, broadening the perspective of the current state of knowledge currently based mostly on bird and mammal models.

## Year-Long Spacing in the Natural Habitat

The spacing patterns of *G. omarorum* in the natural habitat likely reflect year-long territorial defense in both males and females. Territorial defense, usually associated with breeding males, has been proposed to follow two general principles: (1) territory size depends on body size as it is the universal indicator of physical strength and resource holding power (51–53); and (2) territory size depends on individual reproductive state and may be related to circulating androgen levels (54–56). Sexual dimorphism in territory size during breeding can also be expected even in species in which both sexes display territoriality, as males and females may have asymmetries in their motivation and/or their fighting ability. This is the case of red squirrels (*Sciurus vulgaris*), for example, in which males often hold larger territories than females (57) or in the striped plateau lizard (*Sceloporus virgatus*), in which females are more territorial than males (58).

During the breeding season (corresponding to the austral spring-summer, from December to February), this sexually monomorphic species displays similar patterns of spatial arrangement for males and females (59). In resting diurnal conditions, both males and females are found occupying individual spots, distanced at least a meter away from their closest neighbor. A close analysis shows that sex is relevant in spatial arrangements, as animals are more likely to have an opposite-sex than a same-sex closest neighbor. Although males and females hold same-sized territories, when the size of each territory is normalized to its owner's body size, sexual dimorphism arises as females hold relatively larger territories. This interesting difference is probably due to sex-biased reproductive requirements associated to anisogamy, which may lead to higher metabolic requirements in females and thus the need for larger foraging grounds. In male *G. omarorum* gonadosomatic index (GSI) did not show correlation to territory sizes, but circulating 11-ketotestosterone (11-KT, the main bioactive androgen in teleost fish) marginally predicted territory size (59). This data falls in line with the well documented relationship between androgens and male territorial behavior (60–62). In contrast, both female GSI and circulating estradiol show high predicting power on territory size, which constitutes the first report to associate circulating estradiol and territory size in a vertebrate species (59). In the light of the evidence that estradiol promotes female aggression (63–65), ovarian estradiol is likely involved in the modulation of breeding territorial aggression in this species. In summary, during the breeding season, sexually dimorphic individual traits seem to influence motivation toward territory defense in *G. omarorum* impacting on individual spacing in the wild in a sex-dependent manner.

During the non-breeding season (corresponding to the austral autumn-winter, from June to August), adults of *G. omarorum* occupy individual spots in the wild separated at least one meter from the closest neighbor. Sex of individuals does not bias spacing, as closest neighbors are randomly opposite-sex or same-sex. Body size, but not sex, correlates positively with territory size (59). Motivation to maintain territories in the non-breeding season may be related to the fact that these fish continuously produce electric signals as a means of communicating and imaging their world. Electrogeneration is an energetically expensive process which has been associated with high basal metabolic requirements (39, 66) and most likely imposes high year-long foraging demands. Equally sized territories between males and females may reflect the same energetic requirements in both sexes.

## Gonad-Independent Agonistic Behavior Mediates Non-Breeding Territorial Behavior

*G. omarorum* is one of the few teleost species in which the hormonal regulation of non-breeding aggression has been studied [see also damselfish, (22, 27, 67)], and the only teleost species in which the determinants of natural non-breeding spacing have been explored in the field (59).

The acquisition and defense of territories in non-breeding *G. omarorum* have been empirically shown to be mediated by agonistic encounters in laboratory settings (68). When staging dyadic agonistic encounters using a neutral plain arena, all fish engage in rapid escalated conflicts in which the dominant-subordinate status is achieved in <5 min. Subordinates end the struggle when they decide to stop attacking and retreat. In addition, they further signal their surrender electrically: first interrupting their EOD to hide from the dominant, then emitting transient electric submission signals, and finally, adopting a lower post-resolution EOD basal rate (69, 70). The intensity of submission signals emitted by the subordinate individual is correlated to the aggression levels displayed by the dominant (71). Body size is the only predictor of contest outcome, while individual sex has no significant influence (69). After resolution, dominants monopolize the acquired territory and actively exclude subordinate fish to the periphery of the tank (68). Laboratory evidence falls in line with what is observed in the wild, where non-breeding territory sizes are determined by body size and are unrelated to sex. Several pieces of evidence support that the non-breeding agonistic behavior of *G. omarorum* is independent of gonadal hormones. First, intra and intersexual non-breeding agonistic contests are indistinguishable (69, 72). Secondly, aggressive challenges do not have an effect on circulating 11-ketotestosterone (72). Moreover, the clearest evidence of gonadal independence of non-breeding aggression in *G. omarorum* is that agonistic behavior persists unchanged after castration. Gonadectomized and control dyads do not differ in contest outcome, dynamics, aggression levels, nor submissive displays (21), demonstrating that the low levels of non-breeding circulating gonadal hormones are not necessary for the occurrence of this behavior.

## Non-Gonadal Estrogens Modulate Non-Breeding Agonistic Behavior

Brain estrogens are critical regulators of non-breeding aggression. In the absence of high circulating testosterone, brain derived estrogens may be synthesized from circulating adrenal dehydroepiandrosterone (DHEA), proposed to have a key role underlying non-breeding aggression in mammals and birds. DHEA is reported to have higher plasmatic levels in the non-breeding season in birds (26, 73), its levels may respond to social challenges in birds and mammals (26, 74, 75) and it can be metabolized in the brain into active androgens and estrogens (76, 77). In contrast to the breeding season, in non-breeding mammalian and avian models estrogens exert rapid effects upon aggression which reflect non-genomic mechanisms (30, 31, 78, 79). In turn, aggressive interactions can produce changes in steroid hormone levels in specific brain areas of the songbird model (76, 80).

In *G. omarorum* the influence of gonadal hormones in the non-breeding aggression has been ruled out by castration experiments (21), and the role of extra-gonadal steroid hormones has been tested via pharmacological manipulations. Short term involvement of androgens and estrogens was explored focusing on the effects these hormones have on the rapid dynamics of conflict and resolution. Acutely impeding aromatase action by administration of its inhibitor (Fadrozole, 60 min pre contest) in intrasexual dyads had a profound effect in non-breeding agonistic encounters of *G. omarorum*. Overall, results from both male-male and female-female contests show that the inhibition of estrogen synthesis causes a decrease in aggressive displays revealed by an important delay in initiating overt aggression. In addition, it decreases aggression levels and prevents potential winners (larger fish) from achieving dominance (21, 81). Direct short-term effects of androgens were ruled out, since acute treatment with androgen receptor antagonists showed no influence upon conflict engagement, aggression dynamics nor the establishment of dominant-subordinate status (81). If androgens were directly involved in the modulation of non-breeding aggression in *G. omarorum*, their action may be evinced in a longer time frame, as has been observed in other non-breeding territorial fish in which chronic androgen receptor blocking decreases aggression (22).

To date, the expression pattern of brain aromatase has been identified in several teleost species (82–89); including a recent study in the weakly electric fish *Apteronotus leptorhynchus* (90), which also exhibits territorial aggression in non-breeding conditions (43, 91). Aromatase mRNA was mapped in non-breeding male and female *A. leptorhynchus* in the telencephalon, preoptic area, hypothalamus, and pituitary gland, showing a high degree of regional conservation with previous reports in teleosts. Reports of the presence of high levels of aromatase in the social behavior network strongly suggest these neural circuits are affected by local estrogen production. Moreover, testosterone aromatization has reported effects in social behavior electric displays in *Apteronotus* (42, 92). The first transcriptomic study carried out in *G. omarorum* during the non-breeding season shows that aromatase, as well as other steroidogenic enzymes are expressed in the preoptic area (93). This node of the social brain has a well-documented role in aggressive behavior (1, 94–97). Moreover, it has already been shown that preoptic area neuropeptides have a status-dependent role in the modulation of non-breeding aggression in *G. omarorum* (70, 98). The analysis of local brain synthesis of estrogens and androgens in this region regulating non-breeding aggression is currently underway.

Overall, research in *G. omarorum* point to brain estrogen as an important modulator of non-breeding aggression acting in regions of the social brain through rapid mechanisms.

## State of the Art and Perspectives: Neurosteroids Underlying Non-Breeding Aggression

**Figure 1:**
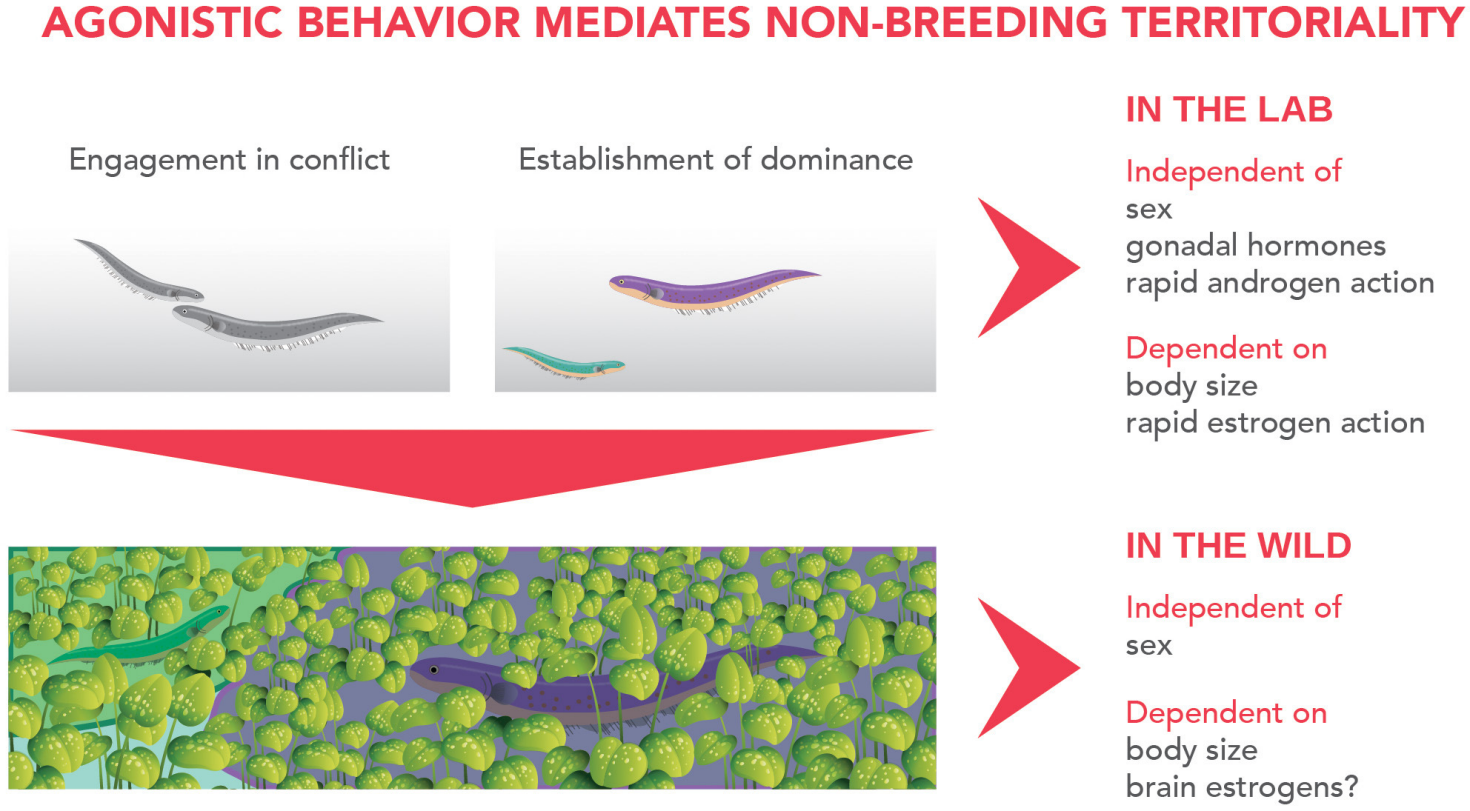
Events and underlying mechanisms of non-breeding territoriality in *Gymnotus omarorum*. Laboratory evidence shows that agonistic behavior mediates territory acquisition, as after conflict resolution dominant animals monopolize the territory and actively exclude subordinate fish to the periphery of the tank. Body size, but not sex, is a strong predictor of conflict outcome. Aggression is maintained even in gonadectomized animals, indicating its independence of gonadal hormones. Behavioral pharmacology evinces aggression is also independent of rapid actions of androgens, although strongly dependent on rapid estrogenic action, as an aromatase inhibitor greatly influences conflict engagement, aggression intensity and establishment of dominance. This evidence suggests that brain-derived estrogens play a key role in agonistic behavior. Agonistic behavior most probably underlies territorial spacing in the natural habitat. Territory sizes are not sex-biased, but do depend on body size, and we propose that at least in the short-term after dominance establishment, they also correlate to brain estrogen levels.

Currently, *G. omarorum* is the strongest teleost model to approach neuroendocrine mechanisms underlying non-breeding aggression. Contributions in this model demonstrate that brain estrogens are key regulators of non-breeding aggression in a much broader sense than previously reported. Revised evidence, brought together from both laboratory and natural settings, shed light on the sequence of events and underlying mechanisms leading to territory acquisition and spatial distribution in the wild (Figure 1). Fish contenders competing for territory display a short evaluation time and engage in escalated conflicts from which a clear dominant-subordinate status emerges. Males and females show no difference in aggressive behavior, but outcome is biased by body size: the larger fish wins and acquires the disputed territory. Agonistic behavior is independent of gonadal hormones and fast acting androgens, although it is strongly dependent on estrogenic action, revealed by the rapid and dramatic effect of blocking estrogen synthesis upon conflict engagement, aggression intensity and establishment of dominance. Agonistic behavior is a key element for the non-breeding distribution of fish in the wild in which animals hold sexually monomorphic territories and body size is the strongest determinant for territory size.

The year-long territorial behavior of *G. omarorum* opens exciting avenues of research on steroid modulation of aggression, and in particular, the yet unexplored role of both circulating and brain-derived steroids in breeding territorial aggression. We have two hypotheses on potential seasonal plasticity in the role of steroids regulating aggression, which are leading our current research. First, we understand that non-breeding contests produce a fast rise in brain estrogen in regions of the social behavior network. This estrogen peak has a rapid, non-genomic effect, promoting aggressive behavior, the fast establishment of dominance, and ultimately, at least in a short time scale, it correlates to the size of the acquired territory in the natural habitat. In absence of high circulating sex steroids, we propose this brain hormonal signature is important in enabling stable territory distributions in natural populations. Secondly, based on the correlation between GSI and territory size in the breeding season, and the independence of aggression from gonads in the non-breeding season, we postulate that regulation of aggression varies seasonally. We hypothesize that estrogens and androgens maintain key roles as modulators, but their main sources alternate from the brain (in the non-breeding season) to the gonads (in the breeding season). In addition, we propose that non-breeding aggression depends exclusively upon brain-derived steroids, either produced *de novo* or from circulating precursors. Studies testing these two hypotheses are underway.

The contributions of *G. omarorum*, a teleost fish with persistent aggression uncoupled from seasonal breeding, expand concepts based on mammal and bird models to further understand the breadth of estrogenic regulation of aggression. Fish are the oldest and most diverse class of vertebrates. Thus, common regulation strategies suggest either a very strong conservation of the trait, or an independent evolution path arriving at the same solution, both underscoring the relevance and extensive impact of estrogens upon aggression.

## Author Contributions

AS and LQ conceived the general organization of the manuscript. AS, LZ, and LQ wrote the manuscript. All authors contributed to the article and approved the submitted version.

## Conflict of Interest

The authors declare that the research was conducted in the absence of any commercial or financial relationships that could be construed as a potential conflict of interest.

## References

[1] Goodson JL Kabelik D. Dynamic limbic networks and social diversity in vertebrates: From neural context to neuromodulatory patterning. Front Neuroendocrinol. (2009) 30:429–41. 10.1016/j.yfrne.2009.05.007 19520105 PMC2763925

[2] Newman SW. The medial extended amygdala in male reproductive behavior A node in the mammalian social behavior network. Ann NY Acad Sci. (1999) 877:242–57. 10.1111/j.1749-6632.1999.tb09271.x 10415653

[3] O'Connell LA Hofmann HA. Genes, hormones, and circuits: An integrative approach to study the evolution of social behavior. Front Neuroendocrinol. (2011) 32:320–35. 10.1016/j.yfrne.2010.12.004 21163292

[4] Kaufmann JH On the definitions and functions of dominance and territoriality. Biol Rev. (1983) 58:1–20. 10.1111/j.1469-185X.1983.tb00379.x

[5] Nelson RJ Biology of Aggression. New York, NY: Oxford University Press (2006).

[6] Wilson EO. Sociobiology: The New Synthesis. Oxford: Belknap Press of Harvard U Press (1975).

[7] Grant JWA Territoriality. Behavioural Ecology of Teleost Fishes. Oxford: Oxford University Press (1997).

[8] Huntingford FA Turner AK Downie LM Animal Conflict. Boca Raton, FL: Chapman & Hall/CRC (1987).

[9] Caldwell G Glickman S Smith E. Seasonal aggression independent of seasonal testosterone in wood rats. Proc Natl Acad Sci USA. (1984) 81:5255–7. 10.1073/pnas.81.16.5255 6591190 PMC391677

[10] Kricher J Behavioral ecology of tropical birds. Wilson Bull. (2001) 113:357–8. 10.1676/0043-5643(2001)113[0357:OL]2.0.CO;2

[11] Wingfield JC Hahn TP Testosterone and territorial behaviour in sedentary and migratory sparrows. Anim Behav. (1994) 47:77–89. 10.1006/anbe.1994.1009

[12] Heimovics SA Trainor BC Soma KK. Rapid effects of estradiol on aggression in birds and mice: the fast and the furious. Integr Comp Biol. (2015) 55:281–93. 10.1093/icb/icv048 25980562 PMC4615795

[13] Munley KM Rendon NM Demas GE. Neural androgen synthesis and aggression: insights from a seasonally breeding rodent. Front Endocrinol. (2018) 9:136. 10.3389/fendo.2018.00136 29670576 PMC5893947

[14] Burger AE Millar RP. Seasonal changes of sexual and territorial behaviour and plasma testosterone levels in male lesser sheathbills (*Chionis minor*). Zeitschrift Tierpsychol. (1980) 52:397–406. 10.1111/j.1439-0310.1980.tb00726.x 7424215

[15] Logan CA Wingfield JC. Autumnal territorial aggression is independent of plasma testosterone in mockingbirds. Hormones Behav. (1990) 24:568–81. 10.1016/0018-506X(90)90042-V 2286368

[16] Schwabl H Kriner E. Territorial aggression and song of male European robins (*Erithacus rubecula*) in autumn and spring: effects of antiandrogen treatment. Hormones Behav. (1991) 25:180–94. 10.1016/0018-506X(91)90049-N 2066079

[17] Gwinner E Rödl T Schwabl H Pair territoriality of wintering stonechats: behaviour, function and hormones. Behav Ecol Sociobiol. (1994) 34:321–7. 10.1007/BF00197002

[18] Soma KK Sullivan K Wingfield J Combined aromatase inhibitor and antiandrogen treatment decreases territorial aggression in a wild songbird during the nonbreeding season. Gen Comp Endocrinol. (1999) 115:442–53. 10.1006/gcen.1999.7334 10480996

[19] Wingfield JC. Regulation of territorial behavior in the sedentary song sparrow, melospiza melodia morphna. Hormones Behav. (1994) 28:1–15. 10.1006/hbeh.1994.1001 8034278

[20] Wingfield JC Lynn S Soma KK. Avoiding the ‘costs' of testosterone: ecological bases of hormone-behavior interactions. Brain Behav Evol. (2001) 57:239–51. 10.1159/000047243 11641561

[21] Jalabert C Quintana L Pessina P Silva A. Extra-gonadal steroids modulate non-breeding territorial aggression in weakly electric fish. Horm Behav. (2015) 72:60–7. 10.1016/j.yhbeh.2015.05.003 25989595

[22] Vullioud P Bshary R Ros AFH. Intra- and interspecific aggression do not modulate androgen levels in dusky gregories, yet male aggression is reduced by an androgen blocker. Horm Behav. (2013) 64:430–8. 10.1016/j.yhbeh.2013.06.007 23838629

[23] Moore MC Marler CA. Effects of testosterone manipulations on nonbreeding season territorial aggression in free-living male lizards, Sceloporus jarrovi. Gen Comp Endocrinol. (1987) 65:225–32. 10.1016/0016-6480(87)90170-5 3817446

[24] Demas GE Cooper MA Albers HE Soma KK Novel mechanisms underlying neuroendocrine regulation of aggression: a synthesis of rodent, avian, and primate studies. In: Handbook of Neurochemistry and Molecular Neurobiology. (2007). p. 337–72.

[25] Jasnow AM Huhman KL Bartness TJ Demas GE. Short-day increases in aggression are inversely related to circulating testosterone concentrations in male Siberian hamsters (Phodopus sungorus). Hormon Behav. (2000) 38:102–10. 10.1006/hbeh.2000.1604 10964524

[26] Hau M Stoddard ST Soma KK. Territorial aggression and hormones during the non-breeding season in a tropical bird. Hormones Behav. (2004) 45:40–9. 10.1016/j.yhbeh.2003.08.002 14733890

[27] Ros AFH Vullioud P Bruintjes R Vallat A Bshary R. Intra- and interspecific challenges modulate cortisol but not androgen levels in a year-round territorial damselfish. J Exp Biol. (2014) 217:1768. 10.1242/jeb.093666 24577440

[28] Soma KK Sullivan KA Tramontin AD Saldanha CJ Schlinger BA Wingfield JC. Acute and chronic effects of an aromatase inhibitor on territorial aggression in breeding and nonbreeding male song sparrows. J Comp Physiol A. (2000) 186:759–69. 10.1007/s003590000129 11016791

[29] Soma KK Tramontin AD Wingfield JC. Oestrogen regulates male aggression in the non-breeding season. Proc Biol Sci. (2000) 267:1089–96. 10.1098/rspb.2000.1113 10885513 PMC1690643

[30] Trainor BC Sima Finy M Nelson RJ. Rapid effects of estradiol on male aggression depend on photoperiod in reproductively non-responsive mice. Hormones Behav. (2008) 53:192–9. 10.1016/j.yhbeh.2007.09.016 17976598 PMC2190085

[31] Trainor BC Lin S Finy MS Rowland MR Nelson RJ. Photoperiod reverses the effects of estrogens on male aggression via genomic and nongenomic pathways. Proc Natl Acad Sci USA. (2007) 104:9840. 10.1073/pnas.0701819104 17525148 PMC1876655

[32] Soma KK Schlinger BA Wingfield JC Saldanha CJ. Brain aromatase, 5α-reductase, and 5β-reductase change seasonally in wild male song sparrows: relationship to aggressive and sexual behavior. J Neurobiol. (2003) 56:209–21. 10.1002/neu.10225 12884261

[33] Soma KK Bindra RK Gee J Wingfield JC Schlinger BA. Androgen-metabolizing enzymes show region-specific changes across the breeding season in the brain of a wild songbird. J Neurobiol. (1999) 41:176–88. 10.1002/(SICI)1097-4695(19991105)41:2<176::AID-NEU2>3.0.CO 10512976

[34] Diotel N Do-Rego J-L Anglade I Vaillant C Pellegrini E Vaudry H. The brain of teleost fish, a source, and a target of sexual steroids. Front Neurosci. (2011) 5:137. 10.3389/fnins.2011.00137 22194715 PMC3242406

[35] Forlano PM Bass AH. Seasonal plasticity of brain aromatase mRNA expression in glia: divergence across sex and vocal phenotypes. J Neurobiol. (2005) 65:37–49. 10.1002/neu.20179 16003720

[36] O'Connell LA Hofmann HA. Evolution of a vertebrate social decision-making network. Science. (2012) 336:1154. 10.1126/science.1218889 22654056

[37] Diotel N Page YL Mouriec K Tong S-K Pellegrini E Vaillant C. Aromatase in the brain of teleost fish: expression, regulation and putative functions. Front Neuroendocrinol. (2010) 31:172–92. 10.1016/j.yfrne.2010.01.003 20116395

[38] Pasmanik M Callard GV. Aromatase and 5α-reductase in the teleost brain, spinal cord, and pituitary gland. Gen Comp Endocrinol. (1985) 60:244–51. 10.1016/0016-6480(85)90320-X 4065533

[39] Markham MR Ban Y McCauley AG Maltby R. Energetics of sensing and communication in electric fish: a blessing and a curse in the anthropocene? Integr Comp Biol. (2016) 56:889–900. 10.1093/icb/icw104 27549201

[40] Bass AH. A hormone-sensitive communication system in an electric fish. J Neurobiol. (1986) 17:131–55. 10.1002/neu.480170303 3519861

[41] Dulka J Maler L Ellis W. Androgen-induced changes in electrocommunicatory behavior are correlated with changes in substance P-like immunoreactivity in the brain of the electric fish Apteronotus leptorhynchus. J Neurosci. (1995) 15:1879. 10.1523/JNEUROSCI.15-03-01879.1995 7534341 PMC6578120

[42] Dulka JG Maler L Testosterone modulates female chirping behavior in the weakly electric fish, Apteronotus leptorhynchus. J Comp Physiol A. (1994) 174:331–43. 10.1007/BF00240215

[43] Dunlap KD. Hormonal and body size correlates of electrocommunication behavior during dydadic interactions in a weakly electric fish, Apteronotus leptorhynchus. Hormones Behav. (2002) 41:187–94. 10.1006/hbeh.2001.1744 11855903

[44] Dunlap KD Chung M Castellano JF. Influence of long-term social interaction on chirping behavior, steroid levels and neurogenesis in weakly electric fish. J Exp Biol. (2013) 216:2434. 10.1242/jeb.082875 23761468 PMC3680506

[45] Gavassa S Goldina A Silva AC Stoddard PK. Behavioral ecology, endocrinology and signal reliability of electric communication. J Exp Biol. (2013) 216:2403. 10.1242/jeb.082255 23761465 PMC3680505

[46] Pouso P Quintana L Bolatto C Silva AC. Brain androgen receptor expression correlates with seasonal changes in the behavior of a weakly electric fish, *Brachyhypopomus gauderio*. Hormones Behav. (2010) 58:729–36. 10.1016/j.yhbeh.2010.07.005 20688071

[47] Smith GT. Evolution and hormonal regulation of sex differences in the electrocommunication behavior of ghost knifefishes (Apteronotidae). J Exp Biol. (2013) 216:2421. 10.1242/jeb.082933 23761467

[48] Zakon H. Weakly electric fish as model systems for studying long-term steroid action on neural circuits. Brain Behav Evol. (1993) 42:242–51. 10.1159/000114158 8252376

[49] Borde M Quintana L Comas V Silva A. Hormone-mediated modulation of the electromotor CPG in pulse-type weakly electric fish. Commonalities and differences across species. Dev Neurobiol. (2020) 80:70–80. 10.1002/dneu.22732 31955508

[50] Black-Cleworth P The role of electrical discharges in the non-reproductive social behaviour of *Gymnotus carapo* (Gymnotidae, Pisces). Anim Behav Monographs. (1970) 3:1–IN1. 10.1016/S0066-1856(70)80001-2

[51] Elliott JM Mechanisms responsible for population regulation in young migratory trout, *Salmo trutta*. III The role of territorial behaviour. J Anim Ecol. (1990) 59:803–18. 10.2307/5015

[52] Keeley ER. An experimental analysis of territory size in juvenile steelhead trout. Anim Behav. (2000) 59:477–90. 10.1006/anbe.1999.1288 10715169

[53] Woodward G Ebenman B Emmerson M Montoya JM Olesen JM Valido A. Body size in ecological networks. Trends Ecol Evol. (2005) 20:402–9. 10.1016/j.tree.2005.04.005 16701403

[54] Adkins-Regan E Hormones and Animal Social Behavior. New Jersey, NJ: Princeton University Press (2005).

[55] Mougeot F Redpath SM Moss R Matthiopoulos J Hudson PJ. Territorial behaviour and population dynamics in red grouse Lagopus lagopus scoticus. I Population experiments. J Anim Ecol. (2003) 72:1073–82. 10.1046/j.1365-2656.2003.00781.x15891828

[56] Watson A Parr R Hormone implants affecting territory size and aggressive and sexual behaviour in red grouse. Scand J Ornithol. (1981) 12:55–61. 10.2307/3675905

[57] Wauters L Dhondt AA Spacing behaviour of red squirrels, *Sciurus vulgaris*: variation between habitats and the sexes. Anim Behav. (1992) 43:297–311. 10.1016/S0003-3472(05)80225-8

[58] Smith DC Home range and territory in the striped plateau lizard. (*Sceloporus virgatus*). Anim Behav. (1985) 33:417–27. 10.1016/S0003-3472(85)80066-X

[59] Zubizarreta L Quintana L Hernández D Teixeira de Mello F Meerhoff M Massaaki Honji R. (2020) Seasonal and social factors associated with spacing in a wild territorial electric fish. PLoS ONE. 15:e0228976. 10.1371/journal.pone.0228976 32542049 PMC7295226

[60] Monaghan EP Glickman SE Hormones and aggressive behavior. Behav Endocrinol. (1992) 134:692–4.

[61] Nelson RJ An Introduction to Behavioral Endocrinology. 3rd ed Sunderland, MA: Sinauer Associates (2005).

[62] Simon NG Lu S-F Androgens and aggression. In: Nelson RJ, editor. Biology of Aggression. New York, NY: Oxford University Press (2006). p. 211–30.

[63] Albert DJ Jonik RH Walsh ML. Hormone-dependent aggression in female rats: testosterone implants attenuate the decline in aggression following ovariectomy. Physiol Behav. (1990) 47:659–64. 10.1016/0031-9384(90)90074-E 2385636

[64] Rendon NM Amez AC Proffitt MR Bauserman ER Demas GE. Aggressive behaviours track transitions in seasonal phenotypes of female Siberian hamsters. Funct Ecol. (2017) 31:1071–81. 10.1111/1365-2435.12816 28757672 PMC5526640

[65] Scaia MF Morandini L Noguera C Trudeau VL Somoza GM Pandolfi M. Can estrogens be considered as key elements of the challenge hypothesis? The case of intrasexual aggression in a cichlid fish. Physiol Behav. (2018) 194:481–90. 10.1016/j.physbeh.2018.06.028 29935215

[66] Stoddard PK Salazar VL. Energetic cost of communication. J Exp Biol. (2011) 214:200. 10.1242/jeb.047910 21177941 PMC3008630

[67] Ros AFH Damjanovic K Glauser G Bshary R. No scope for social modulation of steroid levels in a year-round territorial damselfish. J Exp Zool Part A Ecol Genet Physiol. (2015) 323:80–8. 10.1002/jez.1900 25366877

[68] Perrone R Pedraja F Valiño G Tassino B Silva A Non-breeding territoriality and the effect of territory size on aggression in the weakly electric fish, *Gymnotus omarorum*. Acta Ethol. (2019) 22:79–89. 10.1007/s10211-019-00309-7

[69] Batista G Zubizarreta L Perrone R Silva A Non-sex-biased dominance in a sexually monomorphic electric fish: fight structure and submissive electric signalling. Ethology. (2012) 118:398–410. 10.1111/j.1439-0310.2012.02022.x

[70] Perrone R Silva AC. Status-dependent vasotocin modulation of dominance and subordination in the weakly electric fish *Gymnotus omarorum*. Front Behav Neurosci. (2018) 12:1. 10.3389/fnbeh.2018.00001 29403366 PMC5778121

[71] Zubizarreta L Stoddard PK Silva A Aggression levels affect social interaction in the non-breeding territorial aggression of the weakly electric fish, *Gymnotus omarorum*. Ethology. (2015) 121:8–16. 10.1111/eth.12299

[72] Quintana L Zubizarreta L Jalabert C Batista G Perrone R Silva A. Building the case for a novel teleost model of non-breeding aggression and its neuroendocrine control. J Physiol. (2016) 110:224–32. 10.1016/j.jphysparis.2016.11.009 27915075

[73] Soma KK Wingfield JC. Dehydroepiandrosterone in songbird plasma: seasonal regulation and relationship to territorial aggression. Gen Comp Endocrinol. (2001) 123:144–55. 10.1006/gcen.2001.7657 11482935

[74] Soma KK Rendon NM Boonstra R Albers HE Demas GE. DHEA effects on brain and behavior: insights from comparative studies of aggression. J Steroid Biochem Mol Biol. (2015) 145:261–72. 10.1016/j.jsbmb.2014.05.011 24928552

[75] Rendon NM Rudolph LM Sengelaub DR Demas GE. The agonistic adrenal: melatonin elicits female aggression via regulation of adrenal androgens. Proc R Soc B. (2015) 282:20152080. 10.1098/rspb.2015.2080 26582025 PMC4685819

[76] Heimovics SA Prior NH Ma C Soma KK Rapid effects of an aggressive interaction on dehydroepiandrosterone, testosterone and oestradiol levels in the male song sparrow brain: a seasonal comparison. J Neuroendocrinol. (2016) 28:12345 10.1111/jne.12345 26648568

[77] Schlinger BA Pradhan DS Soma KK. 3β-HSD activates DHEA in the songbird brain. Neurochem Int. (2008) 52:611–20. 10.1016/j.neuint.2007.05.003 17643555 PMC2441539

[78] Heimovics SA Ferris JK Soma KK. Non-invasive administration of 17β-estradiol rapidly increases aggressive behavior in non-breeding, but not breeding, male song sparrows. Hormones Behav. (2015) 69:31–8. 10.1016/j.yhbeh.2014.11.012 25483754

[79] Laredo SA Villalon Landeros R Dooley JC Steinman MQ Orr V Silva AL. Nongenomic effects of estradiol on aggression under short day photoperiods. Hormones Behav. (2013) 64:557–65. 10.1016/j.yhbeh.2013.06.002 23763907 PMC3851015

[80] Charlier TD Newman AEM Heimovics SA Po KWL Saldanha CJ Soma KK. Rapid effects of aggressive interactions on aromatase activity and oestradiol in discrete brain regions of wild male white-crowned sparrows. J Neuroendocrinol. (2011) 23:742–53. 10.1111/j.1365-2826.2011.02170.x 21623961 PMC3135698

[81] Zubizarreta L Silva AC Quintana L. The estrogenic pathway modulates non-breeding female aggression in a teleost fish. Physiol Behav. (2020) 220:112883. 10.1016/j.physbeh.2020.112883 32199998

[82] Dong W Wang L Thornton C Scheffler BE Willett KL. Benzo(a)pyrene decreases brain and ovarian aromatase mRNA expression in *Fundulus heteroclitus*. Aquat Toxicol. (2008) 88:289–300. 10.1016/j.aquatox.2008.05.006 18571745 PMC2530897

[83] Forlano PM Deitcher DL Myers DA Bass AH. Anatomical distribution and cellular basis for high levels of aromatase activity in the brain of teleost fish: aromatase enzyme and mRNA expression identify glia as source. J Neurosci. (2001) 21:8943. 10.1523/JNEUROSCI.21-22-08943.2001 11698605 PMC6762278

[84] Goto-Kazeto R Kight KE Zohar Y Place AR Trant JM. Localization and expression of aromatase mRNA in adult zebrafish. Gen Comp Endocrinol. (2004) 139:72–84. 10.1016/j.ygcen.2004.07.003 15474538

[85] Hallgren S Olsén KH. Effects on guppy brain aromatase activity following short-term steroid and 4-nonylphenol exposures. Environ Toxicol. (2010) 25:261–71. 10.1002/tox.20494 19489062

[86] Jeng S-R Pasquier J Yueh W-S Chen G-R Lee Y-H Dufour S. Differential regulation of the expression of cytochrome P450 aromatase, estrogen and androgen receptor subtypes in the brain–pituitary–ovarian axis of the Japanese eel. (*Anguilla japonica*) reveals steroid dependent and independent mechanisms. Gen Comp Endocrinol. (2012) 175:163–72. 10.1016/j.ygcen.2011.11.005 22107840

[87] Menuet A Pellegrini E Brion F Gueguen M-M Anglade I Pakdel F. Expression and estrogen-dependent regulation of the zebrafish brain aromatase gene. J Comp Neurol. (2005) 485:304–20. 10.1002/cne.20497 15803511

[88] Menuet A Anglade I Le Guevel R Pellegrini E Pakdel F Kah O. Distribution of aromatase mRNA and protein in the brain and pituitary of female rainbow trout: Comparison with estrogen receptor α. J Comp Neurol. (2003) 462:180–93. 10.1002/cne.10726 12794742

[89] Strobl-Mazzulla PH Lethimonier C Gueguen MM Karube M Fernandino JI Yoshizaki G. Brain aromatase (Cyp19A2) and estrogen receptors, in larvae and adult pejerrey fish *Odontesthes bonariensis*: neuroanatomical and functional relations. Gen Comp Endocrinol. (2008) 158:191–201. 10.1016/j.ygcen.2008.07.006 18691594

[90] Shaw K Krahe R. Pattern of aromatase mRNA expression in the brain of a weakly electric fish, *Apteronotus leptorhynchus*. J Chem Neuroanat. (2018) 90:70–9. 10.1016/j.jchemneu.2017.12.009 29288708

[91] Triefenbach FA Zakon HH Changes in signalling during agonistic interactions between male weakly electric knifefish, *Apteronotus leptorhynchus*. Anim Behav. (2008) 75:1263–72. 10.1016/j.anbehav.2007.09.027

[92] Meyer JH Leong M Keller CH. Hormone-induced and maturational changes in electric organ discharges and electroreceptor tuning in the weakly electric fishApteronotus. J Comp Physiol A. (1987) 160:385–94. 10.1007/BF00613028 3572854

[93] Eastman G Valiño G Radío S Young R Quintana L Zakon H. Brain transcriptomics of agonistic behaviour in the weakly electric fish *Gymnotus omarorum*, a wild teleost model of non-breeding aggression. Sci Rep. (2020) 10:9496. 10.1038/s41598-020-66494-9 32528029 PMC7289790

[94] Kelly AM Goodson JL. Social functions of individual vasopressin-oxytocin cell groups in vertebrates: what do we really know? Front Neuroendocrinol. (2014) 35:512–29. 10.1016/j.yfrne.2014.04.005 24813923

[95] Moore FL Lowry CA. Comparative neuroanatomy of vasotocin and vasopressin in amphibians and other vertebrates. Comp Biochem Physiol C. (1998) 119:251–60. 10.1016/S0742-8413(98)00014-0 9826998

[96] Santangelo N Bass AH. New insights into neuropeptide modulation of aggression: field studies of arginine vasotocin in a territorial tropical damselfish. Proc R Soc B Biol Sci. (2006) 273:3085–92. 10.1098/rspb.2006.3683 17015351 PMC1679891

[97] Veenema AH Beiderbeck DI Lukas M Neumann ID. Distinct correlations of vasopressin release within the lateral septum and the bed nucleus of the stria terminalis with the display of intermale aggression. Hormones Behav. (2010) 58:273–81. 10.1016/j.yhbeh.2010.03.006 20298693

[98] Pouso P Radmilovich M Silva A. An immunohistochemical study on the distribution of vasotocin neurons in the brain of two weakly electric fish, *Gymnotus omarorum* and *Brachyhypopomus gauderio*. Tissue Cell. (2017) 49:257–69. 10.1016/j.tice.2017.02.003 28242105

